# The mechanism of lncRNA‐CRNDE in regulating tumour‐associated macrophage M2 polarization and promoting tumour angiogenesis

**DOI:** 10.1111/jcmm.16477

**Published:** 2021-03-20

**Authors:** Chenyang Han, Yi Yang, Yongjia Sheng, Jin Wang, Wenyan Li, Xiaohong Zhou, Li Guo

**Affiliations:** ^1^ Department of pharmacy The Second Affiliated Hospital of Jiaxing University Jiaxing China; ^2^ Department of Center Laboratory The Second Affiliated Hospital of Jiaxing University Jiaxing China

**Keywords:** long non‐coding RNA‐CRNDE, M2 polarization, macrophages, microenvironment, tumour blood vessels

## Abstract

M2 macrophages can promote liver cancer metastasis by promoting tumour angiogenesis; however, the mechanism underlying macrophage polarization has not been completely revealed. In this study, we mainly explored the mechanism underlying long non‐coding RNA‐CRNDE (lncRNA‐CRNDE) in regulating M2 macrophage polarization and promoting liver cancer angiogenesis. The expression of CRNDE was up‐regulated or down‐regulated in THP‐1 cells (CRNDE^‐/‐^‐THP‐1 cells and pcDNA3.1‐CRNDE‐THP‐1). THP‐1 cells were co‐cultured with liver cancer cell line H22, and M2 polarization was induced in THP‐1 by IL‐4/13 to simulate tumour‐induced macrophage polarization. As a result, after CRNDE overexpression, THP‐1 cell viability was up‐regulated, the expression of M2 membrane marker CD163 was up‐regulated, and the proportion of F4/80 + CD163+ cells was also up‐regulated. ELISA assay showed that the expression of M2 markers (including TGF‐β1 and IL‐10) and chemokines (including CCl22 and CCL22) was up‐regulated, and the expression of key signals (including STAT6, JAK‐1, p‐AKT1, and Arg‐1) was also up‐regulated, which were significantly different compared with the control group (Con). In addition, the intervention effect of CRNDE on THP‐1 was consistent between co‐culture with H22 cells and IL‐4/13 induction assay. The induced M2 THP‐1 cells were co‐cultured with HUVEC. As a result, THP‐1 cells with CRNDE overexpression can promote the migration and angiogenesis of HUVEC cells in vitro and simultaneously up‐regulate the expression of Notch1, Dll4 and VEGFR2, indicating that THP‐1 M2 polarization induced by CRNDE could further promote angiogenesis. The H22 cell tumour‐bearing mouse model was constructed, followed by injection of CRNDE anti‐oligosense nucleotides and overexpression plasmids to interfere CRNDE expression in tumour‐bearing tissues. Consequently, down‐regulation of CRNDE could down‐regulate tumour volume, simultaneously down‐regulate the expression of CD163 and CD31 in tissues, decrease the expression of key proteins (including JAK‐1, STAT‐6, p‐STAT6 and p‐AKT1), and down‐regulate the expression of key angiogenesis‐related proteins (including VEGF, Notch1, Dll4 and VEGFR2). In this study, we found that CENDE could indirectly regulate tumour angiogenesis by promoting M2 polarization of macrophages, which is also one of the mechanisms of microenvironmental immune regulation in liver cancer.

## 1 INTRODUCTION

Tumour‐associated macrophages (TAM) play an important role in the regulation of tumour microenvironment. According to different functions and phenotypes, macrophages can be divided into M1 type (classical activated macrophages) and M2 macrophages (alternatively activated macrophages).^1^, ^2^ M2 macrophages (M2‐TAM) are vitally involved in tumour invasion and metastasis. First, M2‐TAM can promote tumour angiogenesis and provide nutrition for tumour growth.^3^ Second, M2‐TAM can promote the invasion and migration of tumour cells by degrading extracellular matrix (ECM). Before tumour metastasis, macrophages are recruited to remote organs to secrete cytokines to improve the tissue microenvironment, thereby providing suitable microenvironment for the distant metastasis and survival of tumour cells.^4^ In addition, M2‐TAM can also secrete TGF‐β, IL‐10, etc to inhibit tumour immunity.^5^, ^6^ In multiple stages of tumour progression, macrophages can exhibit different phenotypes. The formation of tumour microenvironment can promote the transformation of M1 to M2. M1 macrophage is dominant in early tumour tissues, which can inhibit angiogenesis and activate tumour immunity. After tumour progression, M2 macrophage becomes the dominant type in the microenvironment infiltration, and the increased M2 proportion is parallel to the tumour vessel density.^7^, ^8^ Recent studies have demonstrated that the correlation between tumour cells and the microenvironment can be achieved through non‐coding RNA. Liver cancer is a type of malignant tumour with relatively poor prognosis. Similarly, exosomes derived from liver cancer cells can affect tumour microenvironment, supporting for tumour progression.^9^, ^10^

CRNDE currently has few reports on the regulation of tumour microenvironment, so it is necessary to further explore its expression and mechanism in liver cancer, which can provide a new reference for immunotherapy of liver cancer. Therefore, this research starts with the polarization of macrophages, acting CRNDE promotes the M2 type polarization of macrophages and promotes the role and mechanism of tumour angiogenesis.

At present, CRNDE has been rarely investigated in regulating tumour microenvironment. Therefore, it is necessary to further explore its expression and mechanism in liver cancer, which can provide new reference for immunotherapy in liver cancer. To this end, in this study, we investigated the role of mechanism of CRNDE in promoting tumour angiogenesis by promoting macrophage M2 polarization.

## 2 MATERIAL AND METHODS

### 2.1 Construction of THP‐1 cell line and induction of M2 polarization

THP‐1 cells with down‐regulated expression of CRNDE were constructed by shRNA. shRNA‐CRNDE oligo sequence was shown as follows: Forward: gatccGGTGTTAAGTGTGATGCTTCCCTTCCTGTCAGAGGAAGCATCACACTTAACACCTTTTTg; Reverse: aattcAAAAAGGTGTTAAGTGTGATGCTTCCTCTGACAGGGAAGCCDEG. The synthesis of shRNA‐CRNDE and lentivirus packaging were performed by Invitrogen. THP‐1 cells were transfected with shRNA‐CENDE lentivirus to construct cell line with low CRNDE expression, which was defined as THP‐1‐shRNA.

pcDNA3.1‐CRNDE was used to construct THP‐1 cells with CENDE overexpression. The detailed transfection method was shown in the following. The eukaryotic plasmid pcDNA3.1‐CRNDE (Genepharm Biotechnology Co., Ltd., Shanghai, China) was used for CRNDE overexpression. THP‐1 cells were maintained in complete medium at 37°C containing 5% CO_2_ saturated humidity. Cells were passaged every 3‐5d. Cells in logarithmic phase were used for transfection. To be specific, cells were seeded into 6‐well plates, and transfection was performed when cell confluency reached approximately 80%. Before transfection, serum‐containing medium was discarded, and cells were washed with PBS for two times and added 1ml of Opti‐MEM medium (Gibco, the USA). Afterwards, 1.5μg of plasmid and Lipofectamine 2000 were diluted in 100μl Opti‐MEM medium (Gibco, the USA). After incubation and vortex, the mix was added to cells for incubation. After incubation for 6 h, cells were added with fresh complete medium for further incubation. The THP‐1 cell line with CRNDE overexpression was defined as THP‐1‐pcDNA3.1.

M2 polarization of THP‐1 cells was induced by IL‐4/13, or liver cancer cells H22 were co‐cultured with THP‐1 cells. In the induction model, the final concentration of 100ng/ml PMA and 20ng/ml IL4/13 was used to induce M2 polarization of THP‐1 cells. In the liver cancer induction model, H22 cells and THP‐1 cells were co‐cultured in Transwell chambers.

### 2.2 Methods to detect M2 polarization of THP‐1 cells

#### 2.2.1 Viability of THP‐1 cells by CCK‐8 assay

THP‐1 cells were cultured at 37°C in an incubator containing 5% CO_2_ under induction/co‐culture. After co‐culture for 6h, 12h, 24h, 36h and 48 h, cell viability was tested. After replacing the medium without culture medium, 10μl of CCK‐8 reagent was added for staining. After incubating for additional 4 h, the absorbance was measured using a microplate reader at a wavelength of 450nm. The blank medium was used as the control to calculate cell viability (results were shown as %).

#### 2.2.2 The proportion of F4/80 + CD163+ cells by flow cytometry

THP1 cells were induced or co‐cultured for 48h. THP‐1 cells were subsequently collected, washed with PBS for two times and fixed with methanol. Cells were further incubated with 10 μl of FITC‐CD206 and PE‐F4/80 antibodies in dark for 20 min, washed with PBS for two times, and resuspended in 50 μl of liquid. Samples were subjected to flow cytometry (results were shown as %).

#### 2.2.3 CD163 expression changes by immunofluorescence (IF)

THP1 cells were induced or co‐cultured for 48h. After discarding culture medium, THP‐1 cells were collected, washed with PBS for three times, fixed with 4% formaldehyde at room temperature for 0.5h, permeabilized by 0.2% Triton X‐100 for 5 min, washed with PBS for three times and incubated with CD163 monoclonal antibody at 4°C overnight (dilution 1:300, Abcam, Massachusetts, USA). After washing with PBS for two times, samples were incubated with fluorescent secondary antibody, and the slides were subsequently mounted with 95% glycerol and observed under fluorescent microscope.

#### 2.2.4 Expression levels of cytokines (including VEGF, TGF‐β1 and IL‐10) and chemokines (including CCL22 and CCL24) by ESLIA

THP1 cells were induced or co‐cultured for 48h. The supernatant was extracted, centrifuged at 3000r/min for 20min and subjected to ELISA kit (Abcam, Massachusetts, USA) according to the manufacturer's instruction (results were shown as pg/ml).

#### 2.2.5 Expression of key proteins of M2 polarization by Western blot

THP‐1 cells were induced or co‐cultured for 48 h. Cells were subsequently collected, washed with PBS for two times, lysed in RIPA lysis buffer (Beyotime Biotechnology Co., Ltd., Shanghai, China) containing PMSF solution (dilution 1:100) (Beyotime Biotechnology Co., Ltd., Shanghai, China). Cells were lysed on ice for 0.5h after mixing and centrifuged at 12000rpm for 5min. The supernatant was collected to determine protein quantitation by BCA kit (Beyotime Biotechnology Co., Ltd., Shanghai, China). Afterwards, 8%‐12% SDS‐PAGE gels were prepared according to the molecular weight. And 5X loading buffer was added to protein supernatant up to 20μl. After boiling for 8min, protein samples were subjected to electrophoresis at 80V, which was further switched to 120V. After electrophoresis, protein samples were transferred to PVDF membrane at 300mA constant current for 0.5‐2h. The membranes were blocked with 5% skimmed milk for 2h, incubated with proper monoclonal antibodies diluted in TBST (including STAT6, p‐STAT6, JAK‐1, AKT1 and p‐AKT1, dilution 1:300‐500) (Abcam, Massachusetts, USA). Afterwards, the PVDF membranes were washed with TBST for two times and incubated with HRP‐labelled goat anti‐rabbit IgG antibody (dilution 1:2000) (Abcam, Massachusetts, USA). After incubation, the membranes were visualized by chemiluminescence method, and Image‐Pro Plus 6.0 software was used to analyse the optical density. GAPDH was taken as the internal reference, and the results were shown as the comparison between the optical density value of the target protein and the optical density value of the internal reference.

### 2.3 Effects of CRNDE on THP‐1‐induced angiogenesis

After inducing by IL4/13 or co‐culturing with H22 cells for 72h, THP‐1 cells were co‐cultured with HUVEC cells to observe the angiogenesis of HUVEC cells.

#### 2.3.1 Change of HUVEC migration ability by wound‐healing assay

After induction and co‐culture, THP‐1 cells were co‐cultured with HUVEC cells in Transwell chambers. HUVEC cells were inoculated to reach 80% confluency. Afterwards, a straight line was drawn in the middle, followed by co‐culture of THP‐1 cells and HUVEC cells. After 48 hours, the migration of HUVEC cells was observed. The results were processed by Image J software and shown as migration rate (%).

#### 2.3.2 Viability of HUVEC cells by CCK‐8 assay

THP‐1 cells and HUVEC cells were co‐cultured at 37°C in an incubator containing 5% CO_2_. After co‐culture for 0h, 12h, 24h, 36h and 48 h, cell viability was tested. After replacing the medium without culture medium, 10μl of CCK‐8 reagent was added for additional incubation for 4 h. The absorbance was subsequently measured using a microplate reader at a wavelength of 450nm. The blank medium was used as the control to calculate cell viability (results were shown as %).

#### 2.3.3 Blood formation capacity of HUVEC cells by in vitro tube formation assay

After pre‐coating Matrigel into the lower layer of Transwell chambers, HUVEC cells were inoculated and further co‐cultured with THP‐1 after cell adherence, followed by observation of formation of in vitro blood vessels in HUVEC cells after 48h.

#### 2.3.4 Chick embryo chorioallantoic membrane assay

The chick embryo was incubated in a sterile incubator for 72 h at 37.8°C with humidity of 60%. Sterile surgical scissors were used to punch holes in chick embryos, and 6ml of egg white was sucked by using a 10‐ml syringe, followed by injection of THP‐1 cells into the hole. Five chick embryos were set in each group, and the opening was sealed with scotch tape, followed by incubation for 48h. Image‐Pro Plus 6.1 software was used to analyse the area of blood vessels.

#### 2.3.5 Changes of key protein expression by Western blot

THP‐1 cells and HUVEC cells were co‐cultured for 48 h. Then cells were collected, washed with PBS for two times, and lysed in RIPA lysis buffer containing PMSF solution for cell lysis and protein extraction. The detection of protein expression was the same as described above. The dilution of Notch1, Dll‐4 and VEGFR‐2 monoclonal antibody was 1:500 (Abcam, Massachusetts, USA). After antibody incubation, the PVDF membrane was washed with TBST for two times and incubated with HRP‐labelled anti‐rabbit IgG antibody (dilution 1:2000) (Abcam, Massachusetts, USA). After incubation, the membranes were visualized by chemiluminescence method, and Image‐Pro Plus 6.0 software was used to analyse the optical density. GAPDH was taken as the internal reference, and the results were shown as the comparison between the optical density value of the target protein and the optical density value of the internal reference.

### 2.4 Effects of CRNDE on tumour‐bearing mice

H22 cells in the logarithmic phase were collected and washed with PBS for two times. Cell density was adjusted to 5x10^7^/ml and followed by injection of 0.2ml of cell suspension into the armpits of the forelegs of nude mice. Nude mice were fed in a clean environment, followed by observation of growth of nude mice and the formation of solid tumours. Visible subcutaneous solid tumours appeared around 10‐13 days. At this time, mice were divided into Con, anti‐CRNDE and pcDNA3.1‐CRNDE groups. Mice in the Con group were conventionally reared H22 tumour‐bearing ones. Mice in the anti‐CRNDE group were injected with CRNDE anti‐oligosense nucleotides (Sangon Biotech Company, Shanghai, China) to inhibit CRNDE expression. 10 mg/kg anti‐oligosense was prepared with double distilled water. Mice were injected with 0.3ml of prepared anti‐oligosense once a week for four consecutive weeks. Mice in the pcDNA3.1‐CRNDE group was injected with CRNDE overexpression plasmid through tail vein. After anaesthesia by inhalation before injection, each mouse was injected with 15μg of plasmid through tail vein. The plasmid was diluted to 0.2ml with Ringer's lactate once a week for four consecutive weeks. After intervention, mice in the anti‐CRNDE and pcDNA3.1‐CRNDE groups were sacrificed, followed by detection of CRNDE expression in the tumour tissues by RT‐QPCR.

#### 2.4.1 Expression of CD163 and CD31 by immunohistochemistry (IHC)

After mice were killed by carbon dioxide asphyxiation, tumour tissues were fixed with 4% paraformaldehyde, embedded with paraffin and sectioned. The tissue sections were soaked in 1:50 acetone solution for 3 min, dried, soaked in xylene for twice (10 min each), soaked in absolute ethanol for twice (10 min each), soaked in gradient ethanol, rinsed in deionized water for 5 min and rinsed in PBS for 5 min. Antigen retrieval was subsequently performed at 92‐98°C in 0.01 mol/L citrate buffer for 10‐15 min, followed by cooling for 30 min. The sections were treated with 3% hydrogen peroxide for 10‐15 min to eliminate endogenous peroxidase, blocked with 5% BSA at 37°C for 15‐30min. Afterwards, the sections were incubated with CD163 and CD31 monoclonal antibodies (Abcam, Massachusetts, USA) at 37°C for 1‐2h, washed with PBS for two times, reacted with secondary antibody at 37°C for 30min and washed with PBS for 2min. The sections were incubated with dropwise HRP‐labelled avidin at 37°C for 20 min, visualized by DAB staining for 3‐5 min, counterstained with haematoxylin, soaked in gradient ethanol for 5 min, soaked in absolute alcohol for 10 min, soaked in xylene for 10 min, dehydrated and sealed with resin.

#### 2.4.2 Detects key protein expression by Western blot

After carbon dioxide asphyxiation, the tumour tissues were taken from mice and ground with liquid nitrogen, followed by cell lysis and protein extraction using RIPA lysate. The BCA kit was used for protein quantification, and protein expression detection was the same as described above. The expression of M2 polarization key proteins (including STAT6, p‐STAT6, JAK‐1, AKT1 and p‐AKT1) and angiogenesis regulatory proteins (including Notch1, Dll‐4 and VEGFR‐2) was detected.

### 2.5 Statistical analysis

SPSS19.0 software was used for statistical analysis. The measurement data were expressed as mean ± standard deviation ($\bar{x}$±s). One‐way ANOVA was used for comparison between multiple groups, and the SNK test was used for comparison between groups. A *P* <.05 indicated statistical significance.

## 3 RESULTS

### 3.1 Effects of CRNDE on IL4/13‐induced M2 polarization of macrophages

**FIGURE 1:**
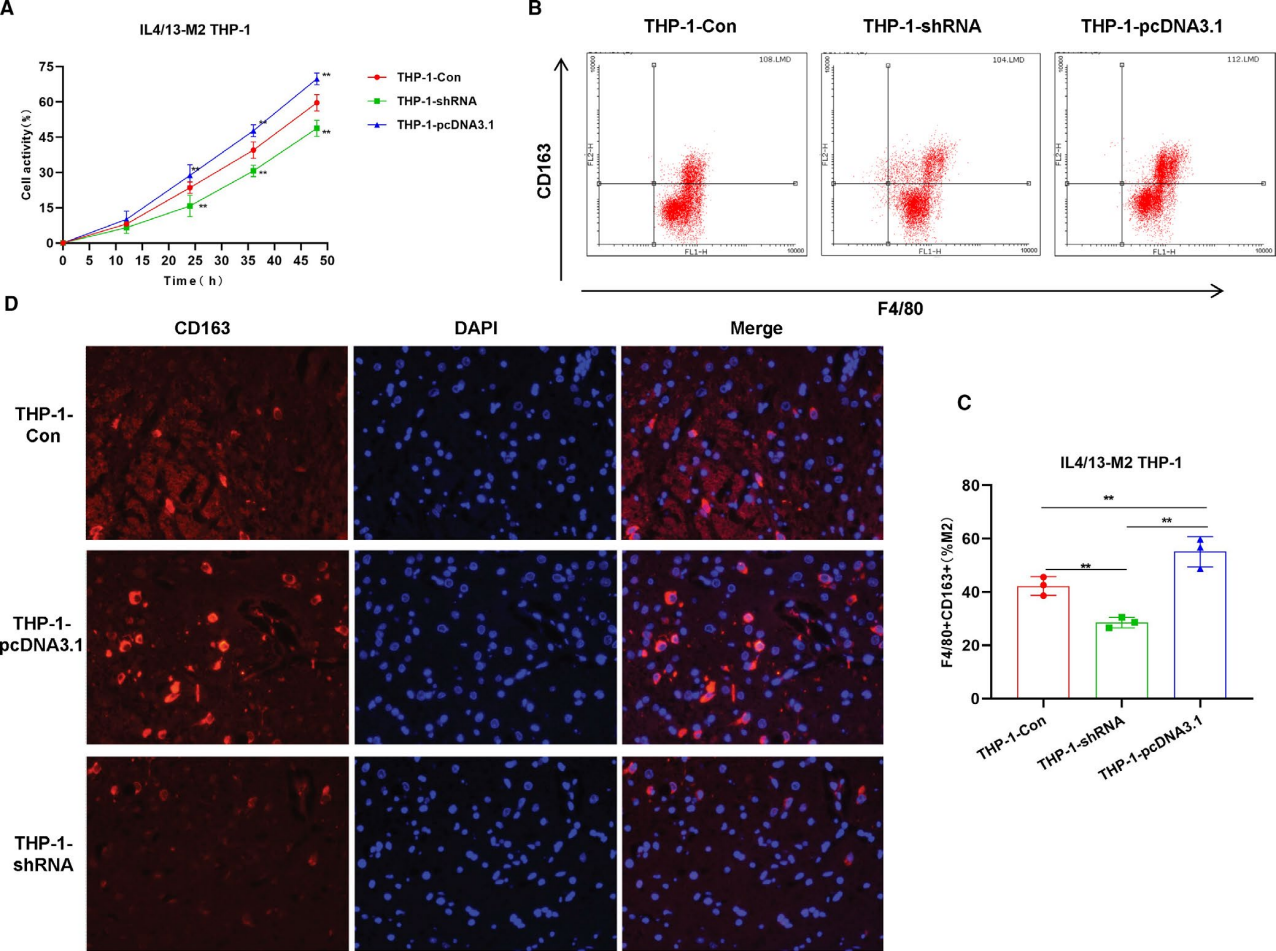
Effects of CRNDE on M2 polarization of THP‐1 cells. A, Cell viability results (n = 5): The cell viability of the THP‐1‐pcDNA3.1 group was significantly higher than that of the THP‐1‐Con group, while the cell viability of the THP‐1‐shRNA group was significantly lower than that of the THP‐1‐Con group. The expression of CRNDE was associated with the viability of THP‐1 cells. Comparison with THP‐1‐Con, *P <.05; **P <.01. B and C, The proportion of F4/80 + CD163+M2 macrophages by flow cytometry (n = 5): The proportion of F4/80 + CD163+M2 macrophages in the THP‐1‐pcDNA3.1 group was significantly higher than that of the THP‐1‐Con group, while the proportion of F4/80 + CD163+M2 macrophages in the THP‐1‐shRNA group was significantly lower than that of the THP‐1‐Con group. Comparison between groups, *P <.05; **P <.01. D, CD163 expression by immunofluorescence staining (n = 3): The expression of CD163 in the THP‐1‐pcDNA3.1 group was significantly higher than that in the THP‐1‐Con group, while the expression of CD163 in the THP‐1‐shRNA group was lower than that of the THP‐1‐Con group

**FIGURE 2:**
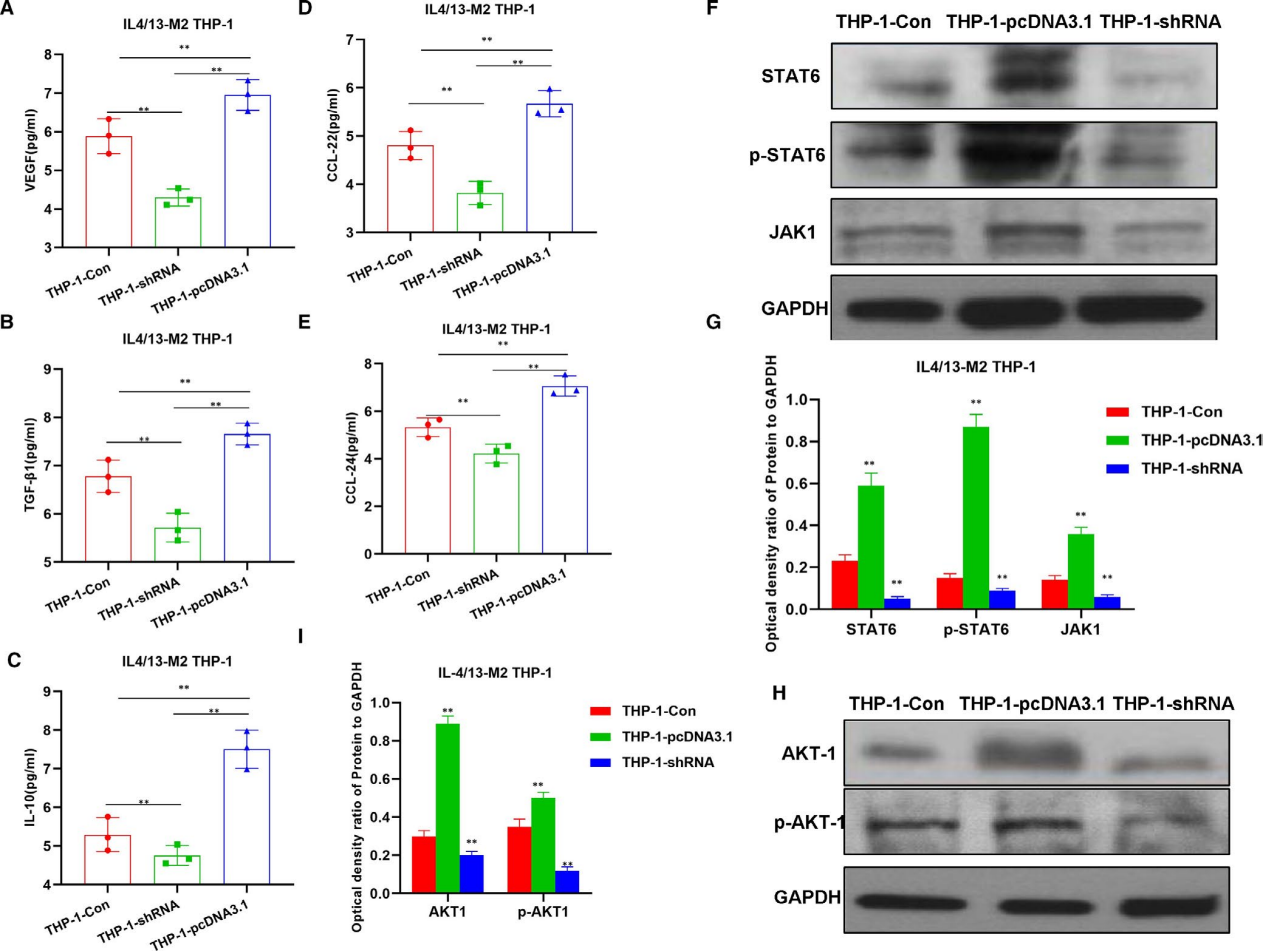
Effects of CRNDE on M2 polarization of THP‐1 cells and the mechanism. A‐E, Detection of M2 macrophage marker expression by CRNDE (n = 5): The expression of M2 macrophage markers in the THP‐1‐pcDNA3.1 group was significantly increased, which was significantly different from the THP‐1‐Con group. Meanwhile, the expression of M2 macrophage markers in THP‐1‐shRNA group was lower than that of the THP‐1‐Con group. Comparison between groups, *P <.05; **P <.01. F‐I, Effects of CRNDE on JAK‐STAT6 and AKT1 signal expression (n = 3): The expression of JAK1, STAT6, p‐STAT6, AKT1 and p‐AKT1 in the THP‐1‐pcDNA3.1 group was significantly higher than that of the THP‐1‐Con group. Meanwhile, the expression level in the THP‐1‐shRNA group was lower than that of the THP‐1‐Con group. Comparison between groups, *P <.05; **P <.01

IL4/13 is commonly used to induce M2 polarization of macrophages. In the study, CRNDE silencing decreased the viability of THP‐1 cells, which was significantly different from the THP‐1‐Con group (*P* <.05). However, the overexpression of CRNDE significantly increased cell vitality, which was significantly higher than the THP‐1‐Con group (*P* <.05), indicating that CENDE was significantly correlated with the vitality of THP‐1 cells (Figure 1A). CD163 is a main marker of M2 macrophages. Flow cytometry was used to detect the proportion of F4/80 + CD163+ cells. As a result, the proportion of F4/80 + CD163+ cells in the THP‐1‐pcDNA3.1 group was significantly higher than that of THP‐1‐Con group, while the proportion of F4/80 + CD163+ cells in the THP‐1‐shRNA group was significantly lower than that of THP‐1‐Con group, indicating that CRNDE affected M2 polarization (Figure 1B,C). IF staining also showed that the expression of CD163 in the THP‐1‐pcDNA3.1 group was significantly higher than THP‐1‐Con group, suggesting that CRNDE affected the expression of CD163. And high expression of CRNDE could promote the expression of CD163, thereby promoting M2 polarization (Figure 1D). In the detection of markers, the expression levels of M2 macrophage markers (including VEGF, IL‐10, TGF‐β1, CCl22 and CCl24) in the THP‐1‐pcDNA3.1 group were significantly up‐regulated, which was higher than the THP‐1‐Con group, while the expression level in the THP‐1‐shRNA group was lower than THP‐1‐Con group, indicating that CRNDE was associated with the expression of markers of M2 macrophages (Figure 2A‐E). JAK1‐STAT6 signal is the main signal of M2 polarization of macrophages and is simultaneously involved in the polarization process. In the detection of protein expression, CRNDE overexpression could further induce the protein expression of JAK1 and STAT6 and promote the phosphorylation of STAT6, while the results were opposite after CRNDE silencing, indicating that CRNDE could promote the activation of JAK1‐STAT6 signal (Figure 2F‐I).

### 3.2 Effects of CRNDE on M2 polarization of macrophages induced by liver cancer cells

**FIGURE 3:**
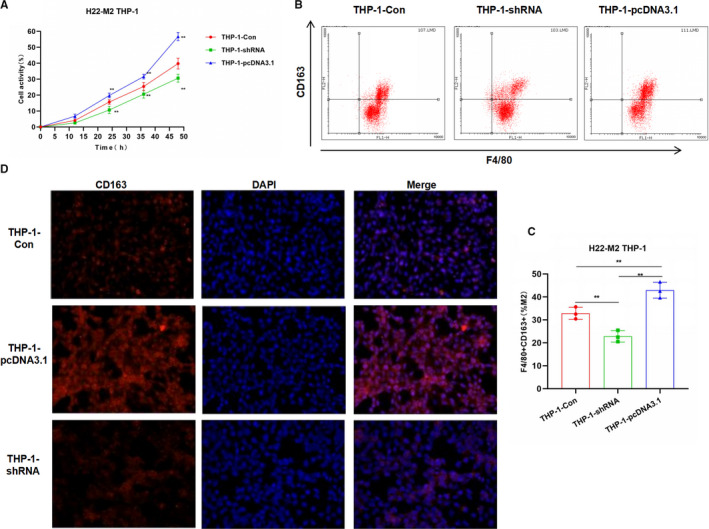
Effects of CRNDE on M2 polarization of THP‐1 induced by liver cancer cells H22. A, Cell viability results (n = 5): The cell viability of the THP‐1‐pcDNA3.1 group was significantly higher than that of the THP‐1‐Con group. The expression of CRNDE was associated with THP‐1 cell viability. Comparison with THP‐1‐Con, *P <.05; **P <.01. B and C, Proportion of F4/80 + CD163+M2 macrophages by flow cytometry (n = 5): The proportion of F4/80 + CD163+M2 macrophages in the THP‐1‐pcDNA3.1 cells was significantly higher than that of THP‐1‐Con group, while the proportion in the THP‐1‐shRNA group was significantly lower than that of THP‐1‐Con group. Comparison between groups, *P <.05; **P <.01. D, CD163 expression by immunofluorescence staining (n = 3): The expression of CD163 in the THP‐1‐pcDNA3.1 group was significantly higher than that in the THP‐1‐Con group, while the expression of CD163 in the THP‐1‐shRNA group was lower than that of the THP‐1‐Con group

**FIGURE 4:**
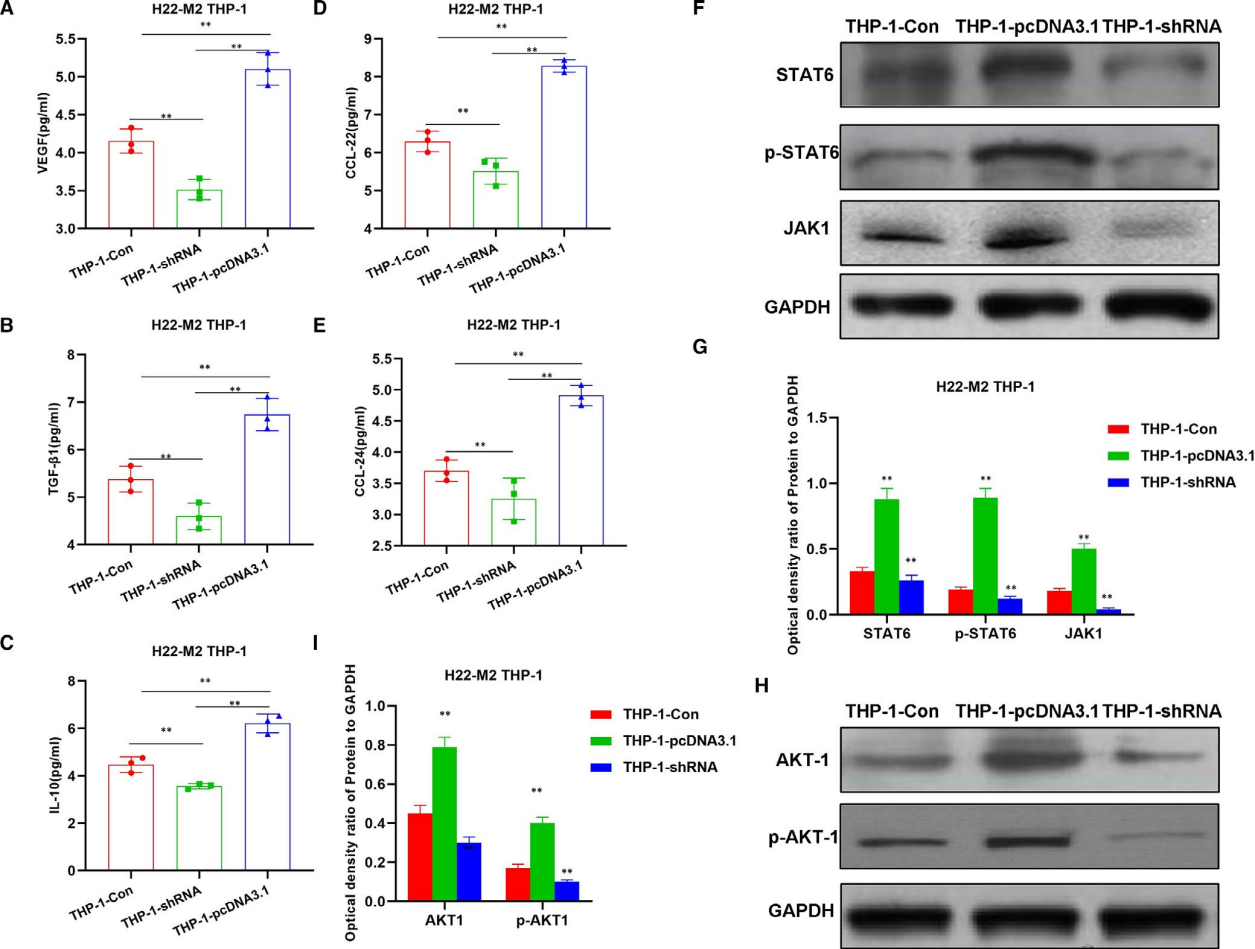
Effects of CENDE on the expression of M2 macrophage markers and key protein expression. A‐E, Detection of M2 macrophage marker expression (n = 5): The expression of M2 macrophage markers was significantly increased in the THP‐1‐pcDNA3.1 group, which was significantly different from the THP‐1‐Con group. Meanwhile, the expression in the THP‐1‐shRNA group was lower than that of THP‐1‐Con group. Comparison between groups, *P <.05; **P <.01. F‐I, Effects of CRNDE on JAK‐STAT6 and AKT1 signal expression (n = 3): The expression of JAK1, STAT6, p‐STAT6, AKT1 and p‐AKT1 in the THP‐1‐pcDNA3.1 group was significantly higher than that of the THP‐1‐Con group. Meanwhile, the expression level in the THP‐1‐shRNA group was lower than that of the THP‐1‐Con group. Comparison between groups, *P <.05; **P <.01

To further verify the tumour‐induced M2 polarization of macrophages, H22 cells were co‐cultured with THP‐1 to investigate the effects of CRNDE on tumour‐induced macrophage polarization. Similar with M2 polarization induced by IL4/13, cell viability was significantly increased after CRNDE overexpression, while cell viability was decreased after CRNDE silencing, which was significantly different from the THP‐1‐Con group (Figure 3A). In the detection of F4/80 + CD163+ cells, the proportion of M2 macrophages in the THP‐1pcDNA3.1 group was significantly higher than that of the THP‐1‐Con, and the proportion of the THP‐1‐shRNA group was lower than that of the THP‐1‐Con (Figure 3B,C). The IF staining of CD163 was significant. To be specific, CRNDE overexpression caused the up‐regulated expression of CD163, while down‐regulation of CRNDE led to decreased expression of CD163 (Figure 3D). In the detection of M2 cell markers, the expression of M2 macrophage markers (including VEGF, IL‐10, TGF‐β1, CCl22 and CCl24) in the THP‐1‐pcDNA3.1 group was significantly higher than that of THP‐1‐Con group, while the expression of M2 macrophage markers in the THP‐1‐shRNA group was lower than that of the THP‐1‐Con group, indicating that CRNDE was associated with M2 macrophage marker expression (Figure 4A‐E). In the detection of the mechanism, the protein expression of JAK1, STAT6 and p‐STAT6 in the THP‐1‐pcDNA3.1 group was significantly higher than that in the THP‐1‐Con group (Figure 4F‐I).

### 3.3 Effects of IL4/13‐induced M2 macrophages on angiogenesis in HUVEC

**FIGURE 5:**
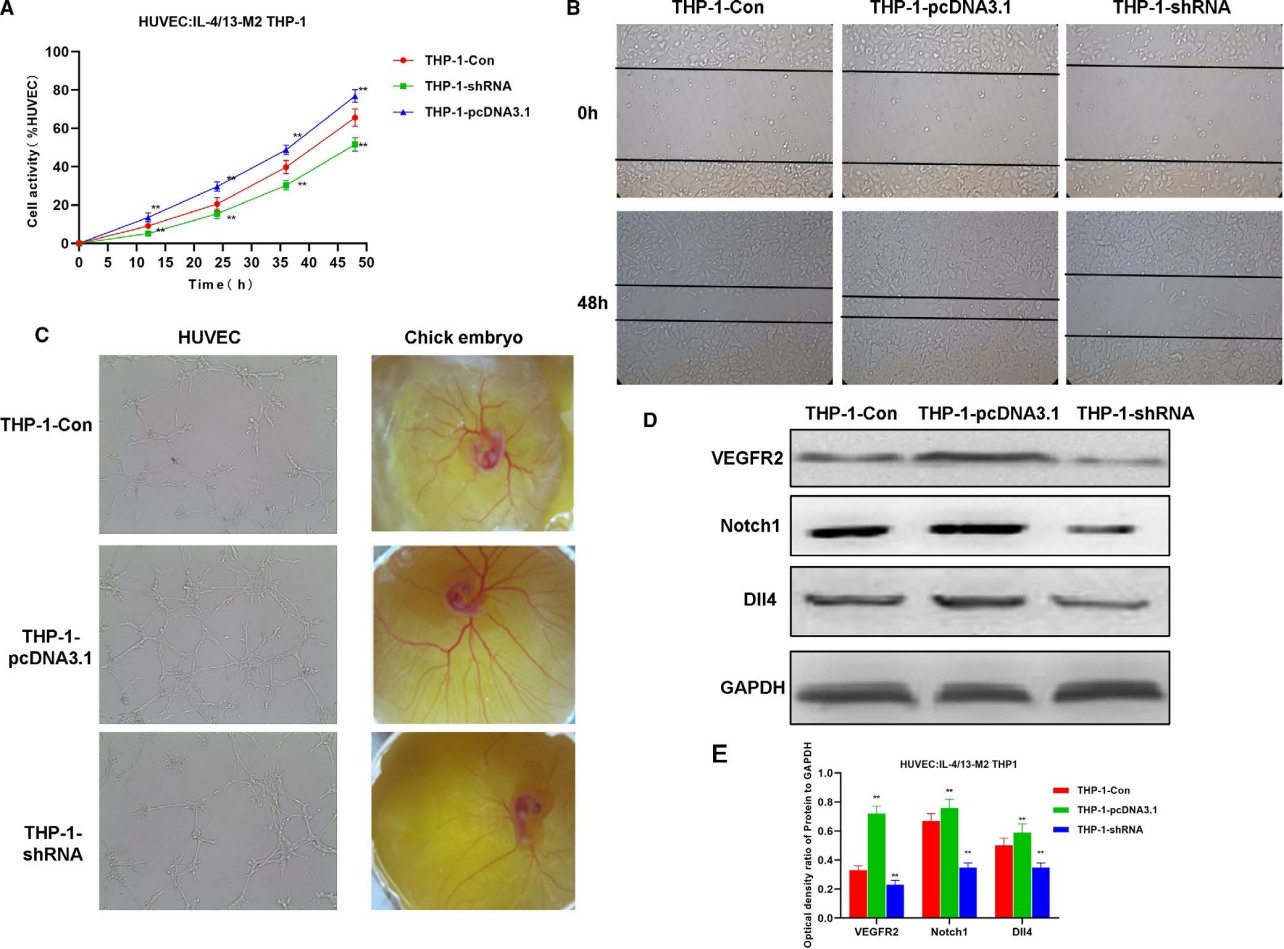
Effects of IL4/13‐induced M2 macrophages on in vitro angiogenesis. A, Effects of M2 macrophages on HUVEC cell viability (n = 5): The cell viability of THP‐1‐pcDNA3.1 group was significantly higher than that of the THP‐1‐Con group. CRNDE expression was associated with THP‐1 cell viability. Comparison with THP‐1‐Con group, *P <.05; **P <.01. B, Results of HUVEC cell migration ability (n = 5): The cell migration ability of the THP‐1‐pcDNA3.1 group was significantly increased than that of the THP‐1‐Con group. C, In vitro tube formation assay of HUVEC and chick embryo chorioallantoic membrane assay (n = 5): the vessel formation of the THP‐1‐pcDNA3.1 group was significantly increased than that of THP‐1‐Con group. D and E, Expression of key signal proteins of angiogenesis (n = 3): The expression of Notch1, Dll4 and VEGFR2 proteins in the THP‐1‐pcDNA3.1 group was significantly higher than that in the THP‐1‐Con group, and the expression in the THP‐1‐shRNA group was lower than the THP‐1‐Con group. Comparison with THP‐1‐Con, *P <.05; **P <.01

M2 macrophages in tumours are an important factor to induce tumour angiogenesis. To further investigate the effects of CRNDE on M2 macrophages‐induced angiogenesis, IL4/13‐induced M2 macrophages were co‐cultured with HUVEC cells. The HUVEC cell viability induced by THP‐1‐pcDNA3.1 group was significantly higher than that of the THP‐1‐Con group, while HUVEC cell viability induced by THP‐1‐shRNA group was lower than that of THP‐1‐Con group (Figure 5A). In the wound‐healing assay, the migration rate of HUVEC cells induced by THP‐1‐pcDNA3.1 group was 79.87 ± 4.55%, while the migration rate of HUVEC cells induced by THP‐1‐Con group was 67.76 ± 5.44%, which was 41.65 ± 8.65% in the THP‐1‐shRNA group (Figure 5B). In vitro vessel formation assay showed that the lumen formation of HUVEC cells induced by the THP‐1‐pcDNA3.1 group, the cell nodes, loops and loop areas were significantly higher than those in the THP‐1‐Con group, while the THP‐1‐shRNA group was lower than that of the THP‐1‐Con group. Chick embryo chorioallantoic membrane assay also showed that THP‐1 cells in the THP‐1‐pcDNA3.1 group could promote blood vessel formation, and the number of capillaries was significantly higher than that in the THP‐1‐Con group. The number of formed blood vessels in THP‐1‐shRNA was significantly lower than that of the THP‐1‐Con group (Figure 5C). The expression of key angiogenesis proteins (including VEGFR2, Notch1 and Dll4) was significantly up‐regulated in the HUVEC induced by THP‐1‐pcDNA3.1, while the expression in the THP‐1‐shRNA group was lower than that in the THP‐1‐Con group (Figure 5D,E).

### 3.4 Effects of M2 macrophages induced by liver cancer cell H22 on angiogenesis in HUVEC

**FIGURE 6:**
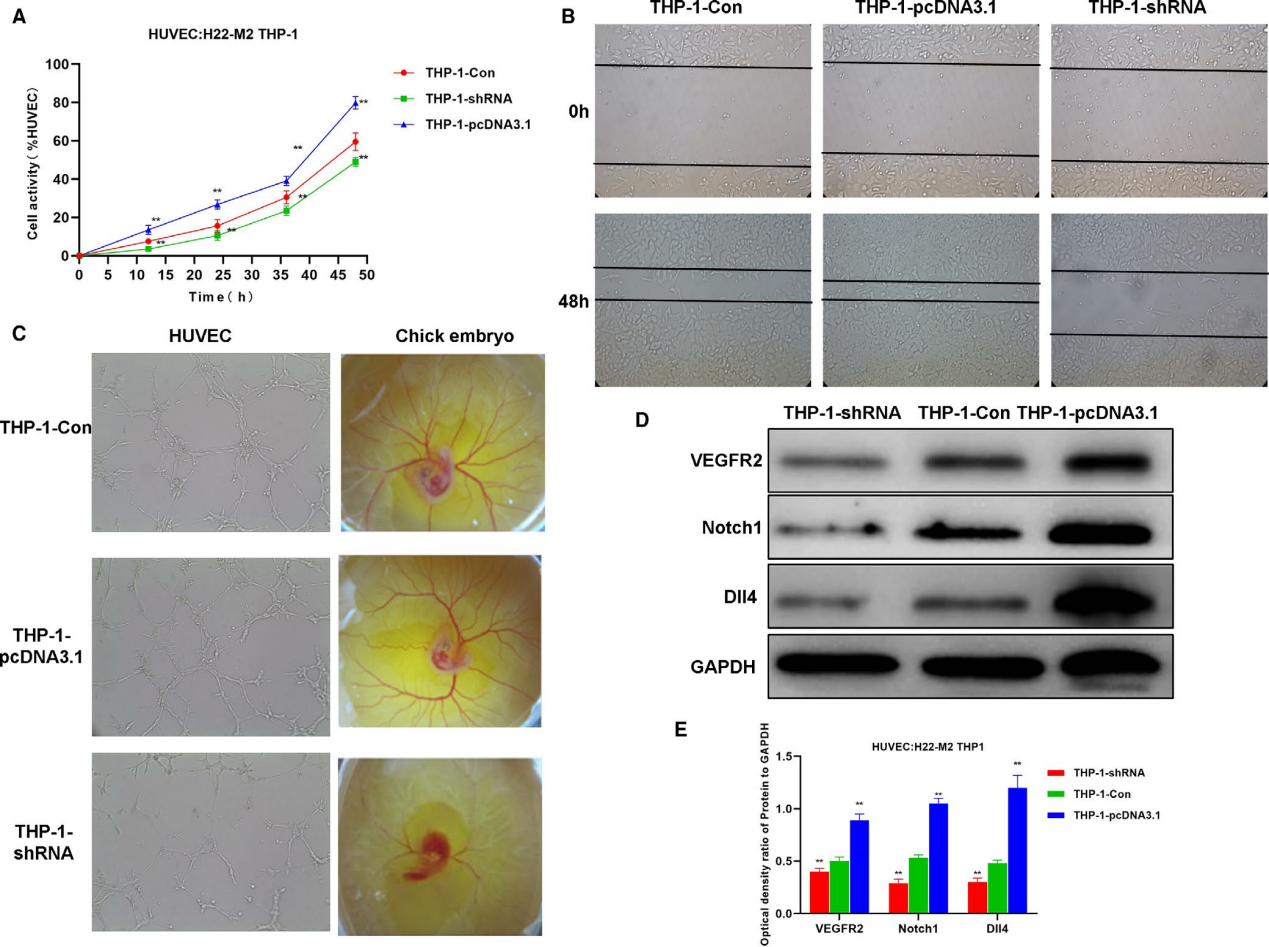
H22‐induced M2 macrophages‐induced angiogenesis. A, HUVEC cell viability results (n = 5): The cell viability of the THP‐1‐pcDNA3.1 group was significantly higher than that of the THP‐1‐Con. Comparison with THP‐1‐Con, *P <.05; **P <.01. B, HUVEC cell migration assay (n = 5): The cell migration capacity of THP‐1‐pcDNA3.1 group was significantly enhanced than that of THP‐1‐Con group. C, In vitro tube formation assay of HUVEC and chick embryo chorioallantoic membrane assay (n = 5): The vessel formation ability of THP‐1‐pcDNA3.1 group was significantly enhanced than THP‐1‐Con group. D and E, Expression of key signal proteins for angiogenesis (n = 3): The protein expression of Notch1, Dll4 and VEGFR2 in the THP‐1‐pcDNA3.1 group was significantly higher than that of the THP‐1‐Con group, and the expression in the HP‐1‐shRNA group was lower than that of the THP‐1‐Con group. Comparison with THP‐1‐Con, *P <.05; **P <.01

Angiogenesis induction assay was performed on M2‐THP‐1 after co‐culture with H22 cells. The results were similar to IL‐4/13‐induced M2 macrophages. The HUVEC cell viability induced by THP‐1‐pcDNA3.1 group was significantly higher than that of THP‐1‐Con group (Figure 6A). The migration rate of HUVEC cells induced by THP‐1‐pcDNA3.1 group was 74.87 ± 8.56%, while the migration rate of HUVEC cells induced by THP‐1‐Con group was 60.16 ± 5.91%, which was 38.34 ± 6.12% in the THP‐1‐shRNA group (Figure 6B). In vitro vessel formation assay showed that the tube formation of HUVEC cells induced by the THP‐1‐pcDNA3.1 group, the cell nodes, loops and loop areas were significantly higher than those in the THP‐1‐Con group. Chick embryo chorioallantoic membrane assay also showed that THP‐1 cells in the THP‐1‐pcDNA3.1 group could promote blood vessel formation, and the number of capillaries was significantly higher than that in the THP‐1‐Con group (Figure 6C). The levels of key angiogenesis protein (including VEGFR2, Notch1 and Dll4) were significantly up‐regulated in HUVEC induced by THP‐1‐pcDNA3.1, while the expression of was lower in THP‐1‐shRNA group than that in THP‐1‐Con group (Figure 6D,E).

### 3.5 Effects of CRNDE on angiogenesis in tumour‐bearing mice

**FIGURE 7:**
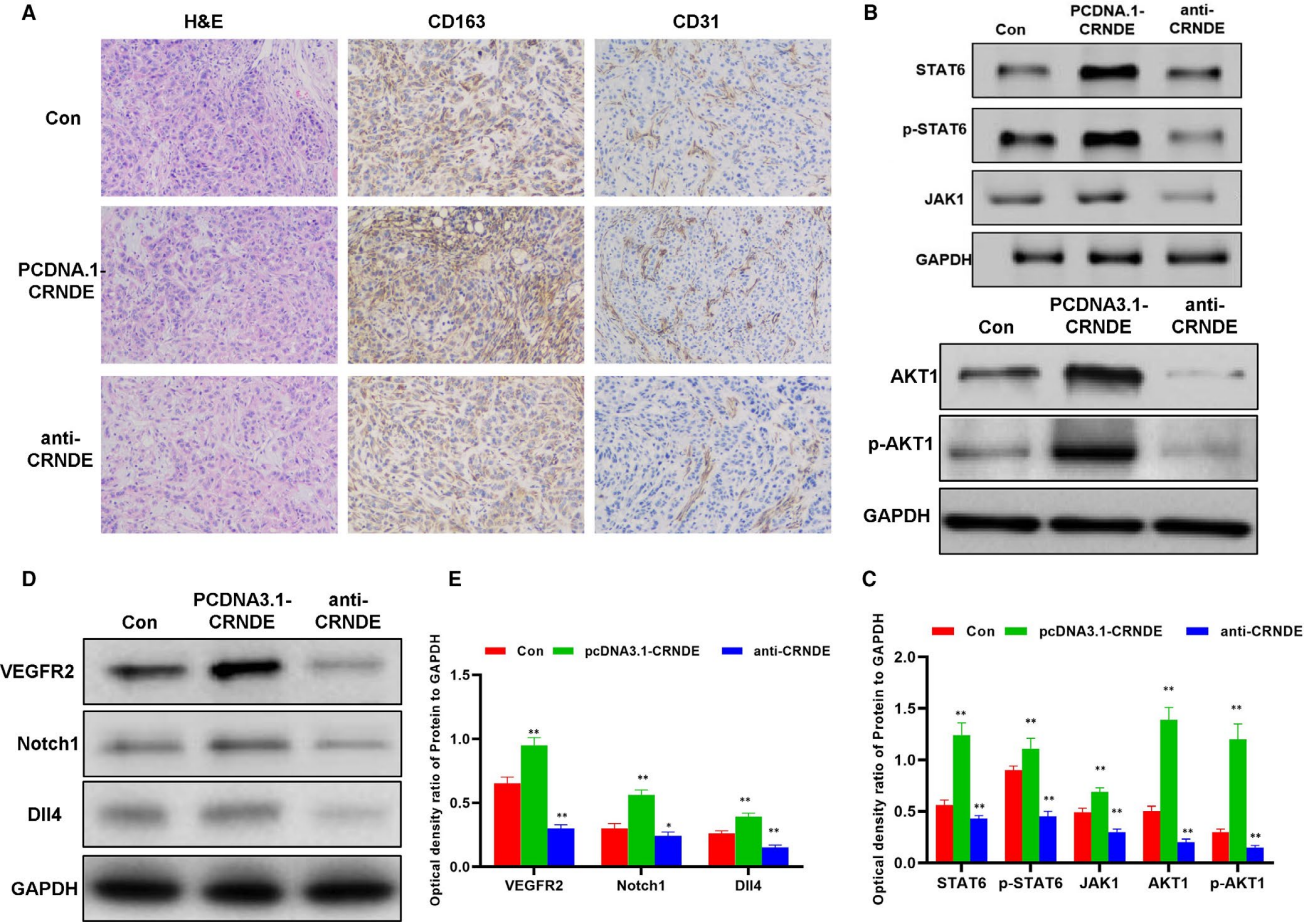
Effects of CRNDE on M2 macrophages and tumour angiogenesis in tumour‐bearing mice. A, Immunohistochemical staining of CD163 and CD31 and H&E staining results of mouse tumour tissues (n = 5): H&E staining showed no obvious tissue lesions among the three groups of mice. The level of CD163 and CD31 in the anti‐CRNDE group was lower than Con group, while the expression of CD163 and CD31 was higher in the pcDNA3.1‐CRNDE group than the Con group. B and C, Expression of key protein of M2 macrophage polarization (n = 3): The protein expression in the pcDNA3.1‐CRNDE group was significantly higher than that in the Con group, while the expression of the anti‐CRNDE group was lower than that of the Con group. Comparison with Con, * *P* <.05; **P <.01. D and E, Expression of key proteins in angiogenesis (n = 3): The protein expression in the pcDNA3.1‐CRNDE group was significantly higher than that in the Con group, while the expression of the anti‐CRNDE group was lower than that of the Con group. Comparison with Con, *P <.05; **P <.01

To further confirm the effects of CRNDE on angiogenesis in mice, liver cancer cell line H22 was used to construct tumour‐bearing mouse model and CRNDE anti‐oligosense was used to inhibit the expression of CRNDE. As a result, the expression of CD163 and CD31 in the tissues of the anti‐CRNDE group was significantly lower than that in the Con group. CD163 is a marker of M2 macrophages, and CD31 is a marker of tumour blood vessels. The down‐regulated expression indicates weaker angiogenesis, which is positively correlated with CD163 (Figure 7A). After the injection of pcDNA3.1‐CRNDE, the overexpression of CRNDE significantly increased the expression of CD163 and CD31. The expression of M2 macrophage key signal proteins (including STAT6, p‐STAT6, JAK1, AKT1 and p‐AKT1) was significantly higher in mouse tissues in the pcDNA3.1‐CRNDE group than that in the Con group (Figure 7B,C). Meanwhile, the expression of angiogenesis‐related protein (including VEGFR2, Notch1 and Dll4) was also up‐regulated (Figure 7D,E).

## 4 DISCUSSION

LncRNA is a type of nucleotide with a transcription length over 200 nt without open reading frame. LncRNAs are able to regulate tumour cell functions. Additionally, lncRNAs can regulate gene expression from different levels such as chromatin recombination, transcription and post‐transcription.^11^, ^12^ CRNDE is a multifunctional lncRNA. Graham et al ^13^ first reported that the expression of CRNDE was up‐regulated in colorectal adenoma and colorectal cancer. CRNDE is barely expressed in normal colon tissues and liver tissues, but increased in colon tissues, rectal cancer, glioma, gastric cancer and liver cancer, suggesting that CRNDE exerts a regulatory effect on tumours.^14^, ^15^ The expression of CRNDE is up‐regulated in human liver cancer tissues and is also associated with clinical staging and lymph node metastasis.^16^ By using RNA sequencing technology to analyse the expression of lncRNA, Esposti found that the expression of CRNDE was up‐regulated, which could be used as a potential diagnostic marker for the prognosis of liver cancer.^17^ Moreover, CRNDE has a regulatory effect on promoting the viability and proliferation of liver cancer cells in vitro and also plays an important role in angiogenesis and tumour growth. Studies have shown that CRNDE can promote the proliferation, migration and invasion of liver cancer cells by inhibiting miRNA‐384 and regulating the expression of NF‐κB and p‐AKT.^18^Our previous study found that miRNA‐136 can target CD163 and inhibit M2 macrophage polarization. It is predicted that CRNDE is the regulatory gene of miRNA‐136, so this is achieved through the action of ceRNA. We use ‘DIANA‐LncBase V2’ database for prediction and verify it by experiments.

Macrophages can be divided into M1 (classically activated) and M2 type (alternatively activated) according to their phenotype and secretory cytokines. The polarization classification of macrophages widely exists in tumours, fats and other tissues and organs, which is also suggestive of prognosis. The common surface markers of M1 macrophages include HLA‐DR, CD197, etc After polarization, M1 macrophages can secrete TNF‐α, iNOS, ROS, etc,^19^ which mainly exert the inflammatory response against microorganisms and play the host immune function, while might simultaneously cause inflammatory damage to normal tissues. Common markers of M2 macrophages include CD163, CD301, etc.^20^ To be specific, F4/80 is a common marker of M1 and M2 macrophages, and multiple indicators are commonly used to distinguish polarization. M2 macrophages mainly secrete IL‐10, VEGF, TGF‐β1, etc to promote wound repair and fibrosis.^21^ In tumour research, M2 macrophages mainly play a role to promote tumour growth and tumour angiogenesis. It is generally believed that M1 macrophages can kill tumour cells while M2 macrophages can promote tumour progression. However, mutual transformation between M1 and M2 type macrophages is common in tumours, which is related to the occurrence and development of tumours. Macrophages play different roles in different stages of tumourigenesis.

The expression of CRNDE in THP‐1 cells was interfered, and IL4/13 was used to induce M2 polarization. The collaborative intervention of IL‐4/13 is a common model of macrophage M2 polarization. As a result, down‐regulation of CRNDE could inhibit THP‐1 cell viability. The effect of macrophages is significantly associated with cell viability. While overexpression of CRNDE could promote the viability of THP‐1, demonstrating that CRNDE plays a vital role on the viability of THP‐1. CRNDE also exerted an effect on the proportion of F4/80 + CD163+M2 macrophages after induction. When CRNDE was overexpressed, the cell proportion was significantly up‐regulated, which was associated with the high expression of CD163. IF staining also showed that CD163 was regulated by CRNDE. In tumour immune research, JNK signalling has been reported to play an important role in M2 polarization. When JNK1 inhibitors are used, the proportion of M2 cells is significantly down‐regulated, and the expression of Arg‐1 and Mrc1 is also down‐regulated. JNK1 plays an important role.^22^ In its downstream STAT receptor, the expression of STAT6 is associated with M2 polarization.^23^ Studies have found that high expression of CRNDE can promote the expression of JNK1, STAT6 and AKT1. PI3K signalling also plays an important role in macrophage polarization mainly by activating downstream AKT1. When CRNDE is overexpressed, JNK‐1, AKT1 and STAT6 are all activated, indicating that CRNDE promotes M2 polarization. H22 cells were co‐cultured with THP‐1 cells to simulate the effect of tumour microenvironment on macrophage polarization. Consequently, overexpression of CRNDE promoted the M2 polarization of THP‐1 and activated the expression of JNK‐1, AKT1 and STAT6. Therefore, through the above experiments, we have confirmed that CRNDE could promote the M2 polarization of THP‐1 cells.

M2 macrophages are mainly associated with tumour angiogenesis. The induced M2‐THP‐1 cells were co‐cultured with HUVEC. As a result, IL‐4/13‐induced M2 macrophages could promote the angiogenesis of HUVEC, and CRNDE overexpression could further promote the formation of blood vessels. H22‐induced THP‐1 cells could also promote the formation of HUVEC, which was similar to that after IL‐4/13 induction. These findings indicated that CRNDE could further promote the formation of tumour blood vessels by inducing macrophages M2 polarization. In vivo experiments in mice also revealed that CRNDE inhibition can decrease the expression of CD163 and CD31. CD31 is a marker of tumour angiogenesis, and down‐regulation of CD31 can reflect the inhibition of vascular proliferation. Therefore, CRNDE is also associated with macrophage M2 polarization and blood vessel formation in the tumour‐bearing mouse model.

## 5 CONCLUSION

In this study, we have found that lncRNA‐CRNDE can promote the M2 polarization of macrophages through the high expression of CD163 to further promote tumour angiogenesis, which is one of the mechanisms by which CRNDE promotes liver cancer progression.

## CONFLICT OF INTEREST

No competing interests.

## AUTHOR CONTRIBUTION

**chenyang han:** Conceptualization (equal); Investigation (equal). **yi yang:** Funding acquisition (equal); Investigation (equal). **yongjia sheng:** Methodology (equal); Software (equal); Visualization (equal). **jin wang:** Investigation (equal); Software (equal); Visualization (equal). **wenyan Li:** Formal analysis (equal); Visualization (equal); Writing‐review & editing (equal). **xiaohong zhou:** Conceptualization (equal); Data curation (equal); Supervision (equal); Writing‐review & editing (equal). **li guo:** Visualization (equal); Writing‐original draft (equal); Writing‐review & editing (equal).

## ETHICAL APPROVAL

The study approvaled with Ethics Committee.

## Data Availability

The data that support the findings of this study are available from the corresponding author upon reasonable request.

## References

[1] Mantovani A , Sozzani S , Locati M , et al. Macrophage polarization: tumor‐associated macrophages as a paradigm for polarized M2 mononuclear phagocytes. Trends Immunol. 2002;23(11):549‐555. 12401408 10.1016/s1471-4906(02)02302-5

[2] Relation T , Yi BT , Guess CAJ , et al. Intratumoral delivery of interferon‐secreting mesenchymal stromal cells repolarizes tumor‐associated macrophages and suppresses neuroblastoma proliferation in vivo. Stem Cells. 2018;36(6):915‐924. 29430789 10.1002/stem.2801

[3] Kawachi A , Yoshida H , Kitano S , et al. Tumor‐associated CD204+ M2 macrophages are unfavorable prognostic indicators in uterine cervical adenocarcinoma. Cancer Sci. 2018;109(3):863‐870. 29274107 10.1111/cas.13476 PMC5834786

[4] Shao X , Wu B , Cheng L , et al. Distinct alterations of CD68+CD163+ M2‐like macrophages and myeloid‐derived suppressor cells in newly diagnosed primary immune thrombocytopenia with or without CR after high‐dose dexamethasone treatment. J Transl Med. 2018;16(1):48‐56. 29499727 10.1186/s12967-018-1424-8 PMC5833082

[5] Han SC , Koo DH , Kang NJ , et al. The generation of Tregs and IL‐10/TGF‐β‐modified macrophages by docosahexaenoic acid via TGF‐β dependent mechanism suppresses atopic dermatitis. Cytokine. 2014;70(1):44.

[6] Huang Z , Gao C , Chi X , et al. IL‐37 Expression is upregulated in patients with tuberculosis and induces macrophages towards an M2‐like phenotype. Scand J Immunol. 2015;82(4):370‐379. 26073153 10.1111/sji.12326

[7] Partecke LI , Günther C , Hagemann S , et al. Induction of M2‐macrophages by tumour cells and tumour growth promotion by M2‐macrophages: A quid pro quo in pancreatic cancer[J]. Pancreatology. 2013;13(5):508‐516. 24075516 10.1016/j.pan.2013.06.010

[8] Palmieri EM , Menga A , Martín‐Pérez R , et al. Pharmacologic or genetic targeting of glutamine synthetase skews macrophages toward an M1‐like phenotype and inhibits tumor metastasis. Cell Rep. 2017;20(7):1654‐1666. 28813676 10.1016/j.celrep.2017.07.054 PMC5575233

[9] Deng Q , Chen S , Fu C , et al. Long noncoding RNA expression profiles in sub‐lethal heat‐treated hepatoma carcinoma cells. World J Surg Oncol. 2017;15(1):136‐146. 28732507 10.1186/s12957-017-1194-4 PMC5521104

[10] Fang J‐H , Zhang Z‐J , Shang L‐R , et al. Hepatoma cell‐secreted exosomal microRNA‐103 increases vascular permeability and promotes metastasis by targeting junction proteins[J]. Hepatology. 2018;68‐75. 10.1002/hep.29920 29637568

[11] Jiao D , Li Z , Zhu M , et al. LncRNA MALAT1 promotes tumor growth and metastasis by targeting miR‐124/foxq1 in bladder transitional cell carcinoma (BTCC)[J]. Am J Cancer Res. 2018;8(4):748‐760. 29736319 PMC5934564

[12] Kong YG , Cui M , Chen SM , et al. LncRNA‐LINC00460 facilitates nasopharyngeal carcinoma tumorigenesis through sponging miR‐149‐5p to up‐regulate IL6. Gene. 2018;639:77‐84. 28987345 10.1016/j.gene.2017.10.006

[13] Ellis BC , Graham LD , Molloy PL . CRNDE, a long non‐coding RNA responsive to insulin/IGF signaling, regulates genes involved in central metabolism. Biochimica et Biophysica Acta (BBA) ‐ Mol Cell Res. 2014;1843(2):372‐386. 10.1016/j.bbamcr.2013.10.016 24184209

[14] Song H , Han LM , Gao Q , et al. Long non‐coding RNA CRNDE promotes tumor growth in medulloblastoma. Eur Rev Med Pharmacol Sci. 2016;20(12):2588‐2595. 27383309

[15] Li Z , Tang Y , Xing W , et al. LncRNA, CRNDE promotes osteosarcoma cell proliferation, invasion and migration by regulating Notch1 signaling and epithelial‐mesenchymal transition. Exp Mol Pathol. 2018;104(1):19‐25. 29246789 10.1016/j.yexmp.2017.12.002

[16] Han S , Han B , Li Z , et al. Downregulation of long noncoding RNA CRNDE suppresses drug resistance of liver cancer cells by increasing microRNA‐33a expression and decreasing HMGA2 expression. Cell Cycle. 2019;2524–2537. 31416393 10.1080/15384101.2019.1652035 PMC6738913

[17] Esposti DD , Hernandezvargas H , Voegele C , et al. Identification of novel long non‐coding RNAs deregulated in hepatocellular carcinoma using RNA‐sequencing. Oncotarget. 2016;7(22):31862‐31877. 26887054 10.18632/oncotarget.7364 PMC5077982

[18] Sun H , He L , Ma L , et al. LncRNA CRNDE promotes cell proliferation, invasion and migration by competitively binding miR‐384 in papillary thyroid cancer. Oncotarget. 2017;8(66):110552‐110565. 29299168 10.18632/oncotarget.22819 PMC5746403

[19] Garg K , Pullen NA , Oskeritzian CA , et al. Macrophage functional polarization (M1/M2) in response to varying fiber and pore dimensions of electrospun scaffolds. Biomaterials. 2013;34(18):4439‐4451. 23515178 10.1016/j.biomaterials.2013.02.065 PMC3623371

[20] Hu JM , Liu K , Liu JH , et al. CD163 as a marker of M2 macrophage, contribute to predicte aggressiveness and prognosis of Kazakh esophageal squamous cell carcinoma. Oncotarget. 2017;8(13):21526‐21538. 28423526 10.18632/oncotarget.15630 PMC5400603

[21] Garg K , Pullen NA , Oskeritzian CA , et al. Modulation, mechanism and angiogenic potential of macrophage polarization (M1/M2) on electrospun bioresorbable vascular grafts. Cardiovascular Pathology. 2013;22(3):e36.

[22] Ma S , Liu M , Xu Z , et al. A double feedback loop mediated by microRNA‐23a/27a/24‐2 regulates M1 versus M2 macrophage polarization and thus regulates cancer progression. Oncotarget. 2015;7(12):13502‐13519. 10.18632/oncotarget.6284 PMC4924657 26540574

[23] Tariq M , Zhang JQ , Liang GK , et al. Gefitinib inhibits M2‐like polarization of tumor‐associated macrophages in Lewis lung cancer by targeting the STAT6 signaling pathway. Acta Pharmacol Sin. 2017;38(11):1501‐1511. 29022575 10.1038/aps.2017.124 PMC5672074

