# Corrigendum

**DOI:** 10.1111/jcmm.17208

**Published:** 2022-03-06

In Chenyang Han et al.,^1^ in Figure 1D and Figure 3D, the fluorescence Figure is inserted low resolution and needs to be corrected. Figure 1B and Figure 3B the flow cytometry deviation in the gate setting operation needs to be corrected. The authors confirm all results and conclusions of this article remain unchanged.

**FIGURE 1:**
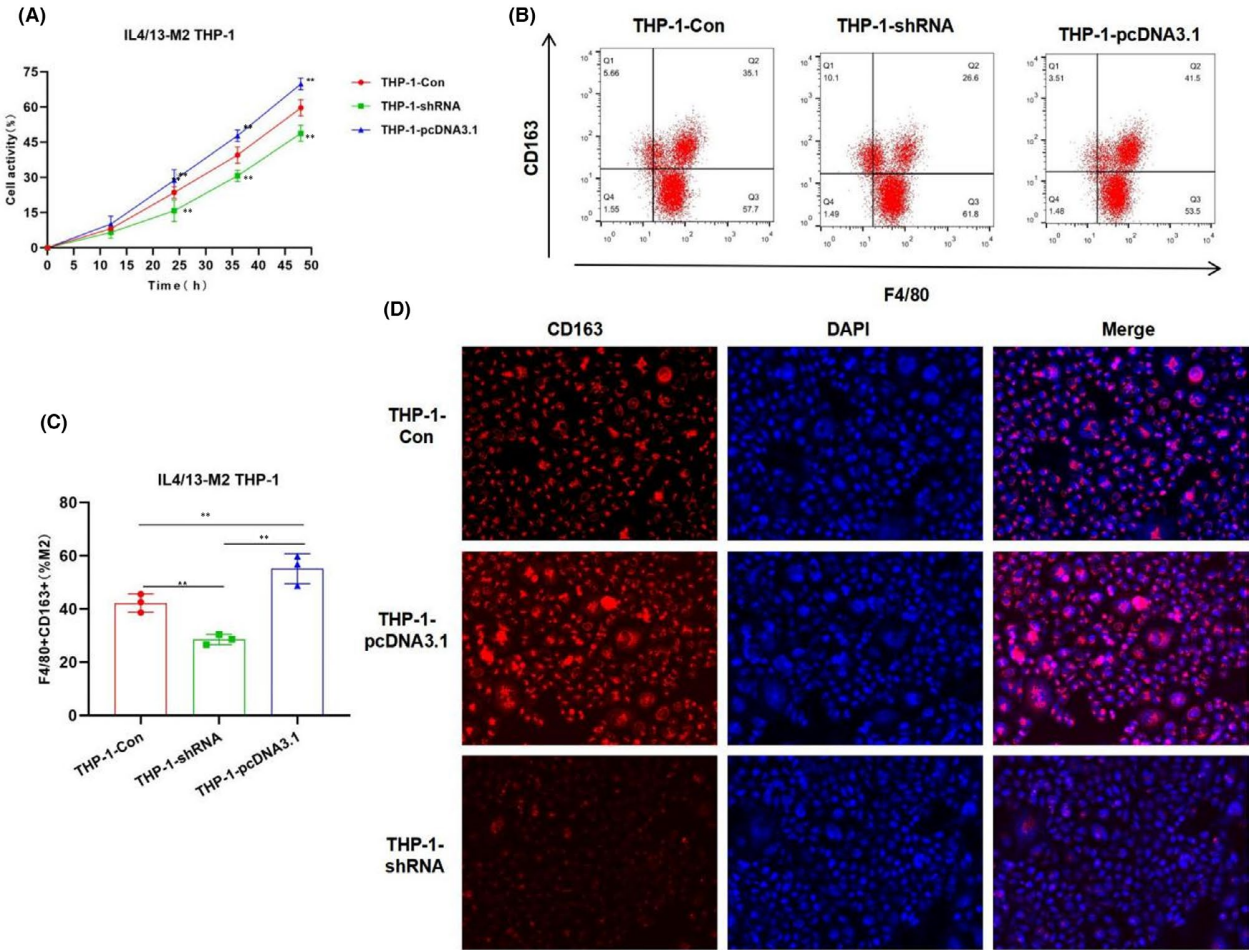
Effects of CRNDE on M2 polarization of THP‐1 cells. (A) Cell viability results (*n* = 5): The cell viability of the THP‐1‐pcDNA3.1 group was significantly higher than that of the THP‐1‐Con group, while the cell viability of the THP‐1‐shRNA group was significantly lower than that of the THP‐1‐Con group. The expression of CRNDE was associated with the viability of THP‐1 cells. Comparison with THP‐1‐Con, *p < 0.05; **p < 0.01. (B and C) The proportion of F4/80 + CD163+M2 macrophages by flow cytometry (*n* = 5): The proportion of F4/80 + CD163+M2 macrophages in the THP‐1‐pcDNA3.1 group was significantly higher than that of the THP‐1‐Con group, while the proportion of F4/80 + CD163+M2 macrophages in the THP‐1‐shRNA group was significantly lower than that of the THP‐1‐Con group. Comparison between groups, *p < 0.05; **p < 0.01. (D) CD163 expression by immunofluorescence staining (*n* = 3): The expression of CD163 in the THP‐1‐pcDNA3.1 group was significantly higher than that in the THP‐1‐Con group, while the expression of CD163 in the THP‐1‐shRNA group was lower than that of the THP‐1‐Con group

**FIGURE 2:**
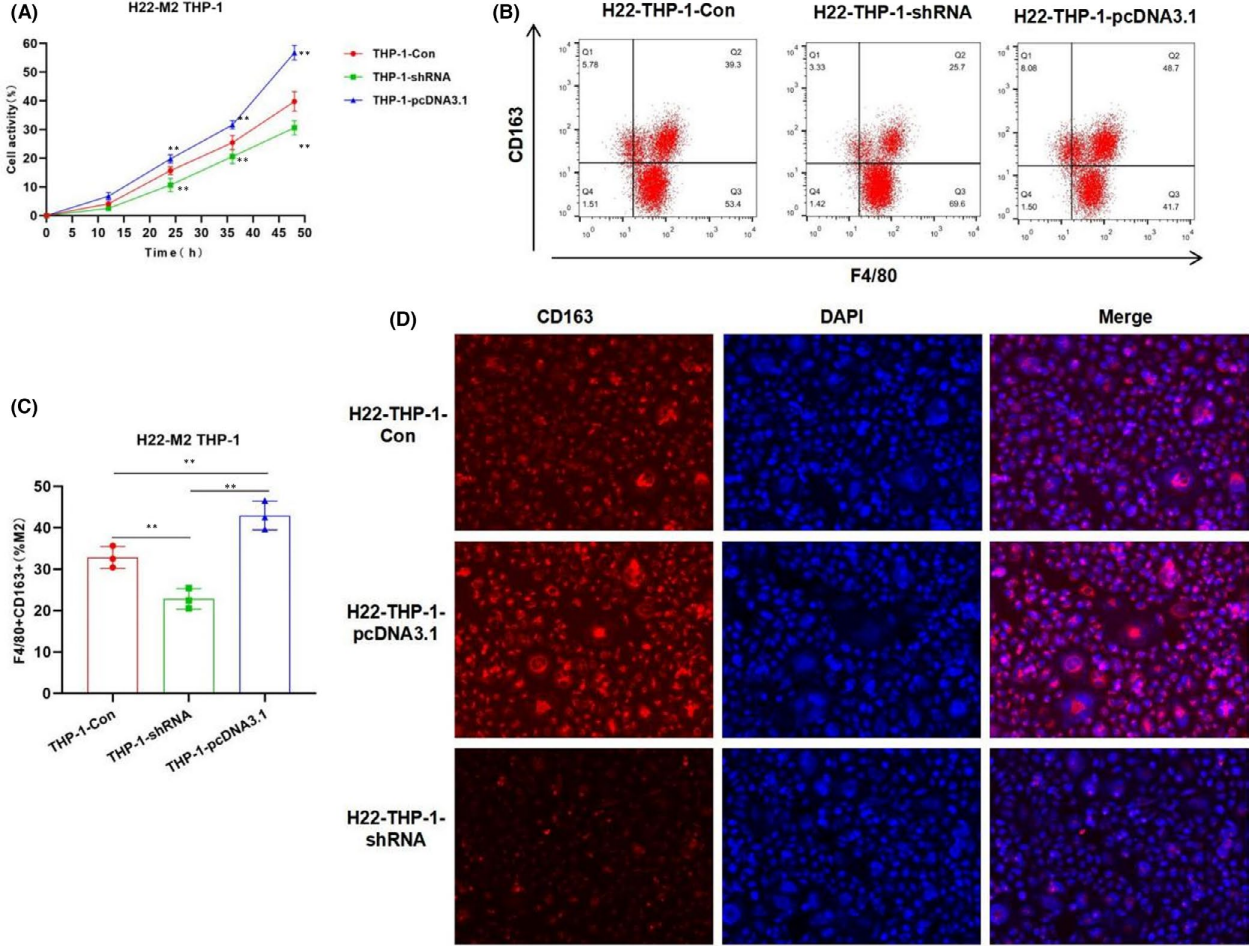
Effects of CRNDE on M2 polarization of THP‐1 induced by liver cancer cells H22. (A) Cell viability results (*n* = 5): The cell viability of the H22‐THP‐1‐pcDNA3.1 group was significantly higher than that of the H22‐THP‐1‐Con group. The expression of CRNDE was associated with H22‐THP‐1 cell viability. Comparison with H22‐THP‐1‐Con, *p < 0.05; **p < 0.01. (B and C) Proportion of F4/80 + CD163+M2 macrophages by flow cytometry (*n* = 5): The proportion of F4/80 + CD163+M2 macrophages in the H22‐THP‐1‐pcDNA3.1 cells was significantly higher than that of H22‐THP‐1‐Con group, while the proportion in the H22‐THP‐1‐shRNA group was significantly lower than that of H22‐THP‐1‐Con group. Comparison between groups, *p < 0.05; **p < 0.01. (D) CD163 expression by immunofluorescence staining (*n* = 3): The expression of CD163 in the H22‐THP‐1‐ pcDNA3.1 group was significantly higher than that in the H22‐THP‐1‐Con group, while the expression of CD163 in the H22‐THP‐1‐shRNA group was lower than that of the H22‐THP‐1‐Con group

## References

[1] Han C , Yang Y , Sheng Y , et al. The mechanism of lncRNA‐CRNDE in regulating tumour‐associated macrophage M2 polarization and promoting tumour angiogenesis. J Cell Mol Med. 2021;25:4235‐4247. 10.1111/jcmm.16477 33742511 PMC8093957

